# Designing a course model for distance-based online bioinformatics training in Africa: The H3ABioNet experience

**DOI:** 10.1371/journal.pcbi.1005715

**Published:** 2017-10-05

**Authors:** Kim T. Gurwitz, Shaun Aron, Sumir Panji, Suresh Maslamoney, Pedro L. Fernandes, David P. Judge, Amel Ghouila, Jean-Baka Domelevo Entfellner, Fatma Z. Guerfali, Colleen Saunders, Ahmed Mansour Alzohairy, Samson P. Salifu, Rehab Ahmed, Ruben Cloete, Jonathan Kayondo, Deogratius Ssemwanga, Nicola Mulder, H3ABioNet Consortium's Education Training and Working Group as members of the H3Africa Consortium

**Affiliations:** 1 Computational Biology Division, Department of Integrative Biomedical Sciences, Institute of Infectious Disease and Molecular Medicine, University of Cape Town, Cape Town, South Africa; 2 Sydney Brenner Institute for Molecular Bioscience, University of the Witwatersrand, Johannesburg, South Africa; 3 Instituto Gulbenkian de Ciência, Oeiras, Portugal; 4 Independent bioinformatics training specialist, Cambridge, United Kingdom; 5 Institut Pasteur de Tunis, LR11IPT02, Laboratory Transmission, Control and Immunobiology of Infections (LTCII), Tunis-Belvédère, Tunisia; 6 South African National Bioinformatics Institute, Medical Research Council Unit for Bioinformatics Capacity Development, University of the Western Cape, Bellville, South Africa; 7 Computer Science Department, University of the Western Cape, Bellville, South Africa; 8 Genetics Department, Faculty of Agriculture, Zagazig University, Zagazig, Egypt; 9 Department of Biochemistry and Biotechnology, Kumasi Centre for Collaborative Research, Kwame Nkrumah University of Science and Technology, Kumasi, Ghana; 10 Centre for Bioinformatics and Systems Biology, Faculty of Science, University of Khartoum, Khartoum, Sudan; 11 Future University of Sudan, Khartoum, Sudan; 12 Uganda Virus Research Institute, Entebbe, Uganda; 13 Uganda Research Unit on AIDS, Medical Research Council/ Uganda Virus Research Institute, Entebbe, Uganda; Genome Quebec, CANADA

## Abstract

Africa is not unique in its need for basic bioinformatics training for individuals from a diverse range of academic backgrounds. However, particular logistical challenges in Africa, most notably access to bioinformatics expertise and internet stability, must be addressed in order to meet this need on the continent. H3ABioNet (www.h3abionet.org), the Pan African Bioinformatics Network for H3Africa, has therefore developed an innovative, free-of-charge “Introduction to Bioinformatics” course, taking these challenges into account as part of its educational efforts to provide on-site training and develop local expertise inside its network. A multiple-delivery–mode learning model was selected for this 3-month course in order to increase access to (mostly) African, expert bioinformatics trainers. The content of the course was developed to include a range of fundamental bioinformatics topics at the introductory level. For the first iteration of the course (2016), classrooms with a total of 364 enrolled participants were hosted at 20 institutions across 10 African countries. To ensure that classroom success did not depend on stable internet, trainers pre-recorded their lectures, and classrooms downloaded and watched these locally during biweekly contact sessions. The trainers were available via video conferencing to take questions during contact sessions, as well as via online “question and discussion” forums outside of contact session time. This learning model, developed for a resource-limited setting, could easily be adapted to other settings.

This is a *PLOS Computational Biology* Education paper.

## Introduction

It is widely accepted that biological analyses are increasingly reliant on computing technologies and that there is a need to train scientists in (at least) the basics of how to utilize computational tools and resources in the analysis of their data [1]. Various training initiatives have provided such introductory training, such as the European Bioinformatics Institute (EMBL-EBI) (http://www.ebi.ac.uk/training), bioinformatics.ca at the Ontario Institute for Cancer Research (OICR) (https://bioinformatics.ca/), and the Gulbenkian Training Programme in Bioinformatics (GBTP) at Instituto Gulbenkian de Ciência (http://gtpb.igc.gulbenkian.pt/bicourses/index.html). These have typically relied on features such as access to expert bioinformatics trainers, infrastructure for training, and stable internet connectivity.

In resource-limited settings, however, specific challenges need to be addressed in order to adequately provide bioinformatics training. Challenges include a lack of access to local bioinformatics expertise, a lack of access to bioinformatics training, and internet access instability [2]. In the past, these challenges have been addressed by physically moving participants to where the resources are located; that is to say, by holding face-to-face training workshops in established, relatively well-resourced locations. A major focus area of H3ABioNet, the Pan African Bioinformatics Network for H3Africa [3], has been to develop sustainable approaches to develop bioinformatics capacity in Africa. Taking into consideration the challenges mentioned above, H3ABioNet has explored various training approaches, including long and short face-to-face training workshops, internships, and data-centered hackathons. Although face-to-face training workshops have proven successful, the model does present several challenges. The workshops are expensive to run, considering the need to finance travel, accommodation, and meals for both the trainers and the participants, which results in a restriction on the number of participants recruited to attend the workshops. In addition, participants may not have access to the infrastructure required to use the skills learned once they return to their home institutes. Furthermore, there are logistical concerns associated with travel, especially in areas not well-serviced by flight routes and the often tedious visa application processes. Finally, it may be disruptive and counterproductive for participants to leave their home institutes for extended periods of time. These challenges raise the question of the true value of face-to-face short courses in terms of what is actually assimilated and learned in the allotted time and what may be disseminated and utilized by the participants at their home institutes.

In an attempt to overcome some of the challenges presented by face-to-face training in a resource-limited environment, one may look to various alternative learning models, which have been used to deliver training courses. For example, distance learning, in which the trainer and participants are not in the same physical location, has been employed by universities for many years in order to open up access to learning in the form of correspondence courses [4]. Ensuring that content may be used as open educational resources (OER) further opens access, especially with advances in technology, in which the internet has been utilized to facilitate the dissemination of these materials. Distance learning and OER indisputably increase access to education; however, both, in their simplest forms, offer only sparse human contact. Students still enjoy, and perhaps prefer, face-to-face learning—having someone to rely on and feel accountable to [5]. In most instances, this preference for face-to-face learning has been attributed to the value of interaction and the presence and opinions of classmates and lecturers, structured time allocated to complete tasks, and immediate feedback to comments or questions raised. In an effort to provide the “best of both worlds,” one might employ a multiple-delivery–mode learning approach, in which elements of various learning models are combined to suit one’s specific training needs [6,7].

H3ABioNet has therefore developed a multiple-delivery–mode learning model comprising elements of distance learning, OER, and face-to-face learning for an “Introduction to Bioinformatics” (IBT) course in order to meet the need for bioinformatics training of molecular biologists—as well as individuals from other backgrounds interested in developing skills in bioinformatics—in Africa and to address the specific challenges for this setting. Below we discuss the course curriculum and key features of our learning model. We highlight the approach used for the 2016 iteration of the course in order to illustrate the logistical setup and the lessons learnt.

## Skills-based course curriculum

The IBT course curriculum comprised an introduction to various bioinformatics topics that H3ABioNet’s Education and Training Working Group identified as important for molecular biologists in African research institutions who would like to become basic “bioinformatics users” (as described by the International Society for Computational Biology’s [ISCB] core bioinformatics competencies [8]). Modules include the following: an introduction to bioinformatics, bioinformatics databases, and resources; genomics; Linux; sequence alignment; protein structural bioinformatics; and phylogenetics (http://training.h3abionet.org/IBT_2016/?page_id=273). These priority topics were selected as essential for African scientists who often face population-level epidemic threats and hence need to develop skills in using bioinformatics resources to rapidly tackle the local global health challenges. Pitching the course at the introductory level helped address an important issue in the African context: many of the current bioinformatics courses are aimed at the advanced researcher level. An introductory-level course is important to equip novices with enough skills and confidence to apply for more advanced courses if they would like to further develop their bioinformatics skills.

Great effort was taken to ensure that the content delivered was relevant, up to date, of a high standard, and developed using sound educational theory. Specific attention was given to differentiating between learning objectives and learning outcomes (S1 File) [9]. Furthermore, each session was designed to be skills based with an emphasis on what the participants will be able to do and which bioinformatics skills they will have acquired after the sessions. All resources—lecture recordings, lecture slides, and practical assignment instructions—remain publicly available under a Creative Commons license on the course website (http://training.h3abionet.org/IBT_2016/).

## H3ABioNet’s multiple-delivery–mode learning model

A multiple-delivery–mode learning model was constructed for our IBT course in order to address the various challenges associated with bioinformatics training in a resource-limited setting, as discussed above. Most notably, the model allows for increased access to information, to local academic and administrative support, and to the tools and course material even after the training has concluded. The contribution of each learning model to our approach is explained below and is summarized in Table 1.

**Table 1:** Components of and rationale behind H3ABioNet’s multiple-delivery–mode learning model for the IBT course.

Challenges in Africa	Learning Model Utilized to Meet These Challenges	Implementation of Learning Model for the IBT Course
Need for local academic and administrative support	Face-to-face learning	Local classrooms with volunteer staff
Lack of access to local bioinformatics expertise and bioinformatics training	Distance learning	Local classrooms connect to expert trainers in a virtual classroom via the Mconf^a^ video conferencing platform and via online forums in Vula^b^—the course management platform
Internet access instability	Distance learning	Pre-recorded lectures and online forums
Lack of access to training materials	OER	Resources publicly available on course website
Coordination of course administration for large cohort (364 enrolled participants)	Distance learning	Practical assignments and assessments administered via Vula
Limited access to software	OER	Web-based, open-source bioinformatics tools are used

^a^ http://mconf.org; hosted by the CSIR SANREN, https://mconf.sanren.ac.za/)

^b^ http://vula.uct.ac.za

Abbreviations: CSIR, Council for Scientific and Industrial Research; H3ABioNet, Pan African Bioinformatics Network for H3Africa; IBT, Introduction to Bioinformatics; OER, open educational resources; SANREN, South African National Research Network.

### Face-to-face learning

Face-to-face learning allowed for local academic and administrative support.

#### Local classrooms

Participating institutions set up local classrooms after certain infrastructure requirements were confirmed, namely, the following: enough computers with internet access for each participant; a projector system to screen the virtual classroom interface and pre-recorded lectures (see section entitled “Distance learning”); a system administrator to provide technical support; and teaching assistants to provide local academic and administrative support. The classroom environment provided the opportunity for participants to discuss bioinformatics concepts with their classmates and teaching assistants and to seek clarification when necessary. It created a support structure that intended to motivate participants and hold them accountable to attending class and to keeping up to date with the course content. This structure also provided a streamlined channel of communication linking the course’s core team to the various classrooms. It is important to mention that this course was offered free of charge to participating institutions and to participants in order to ensure that cost was not a barrier to education.

### Distance learning

Distance learning allowed for access to bioinformatics expertise and training.

#### Virtual classroom

**Fig 1:**
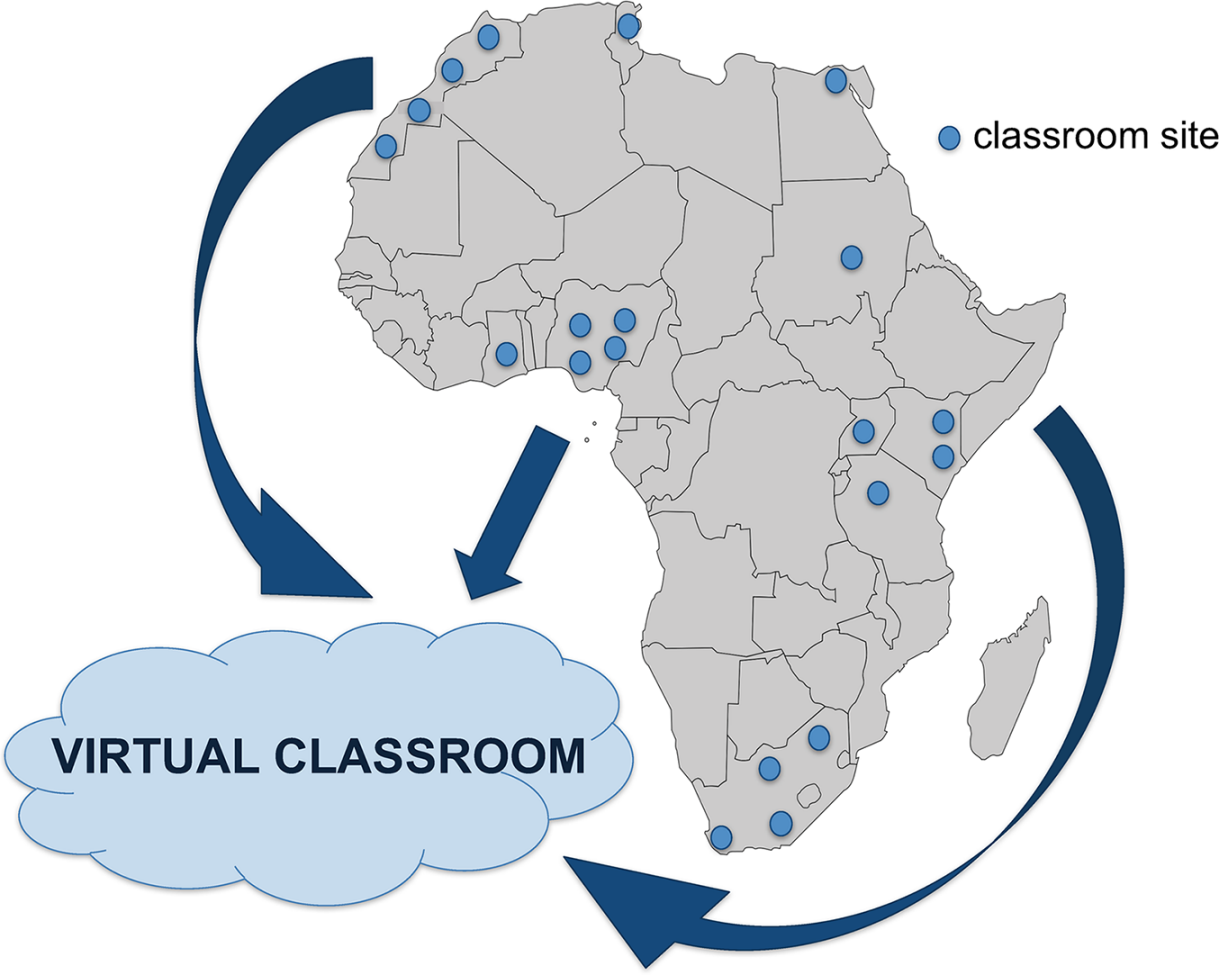
Location of classroom sites for 2016 iteration of the IBT course. Classrooms (20 classrooms across 10 African countries accommodating 364 participants in total) met each other virtually to widen the scope and improve the depth of biweekly contact sessions. Abbreviation: IBT, Introduction to Bioinformatics.

**Fig 2:**
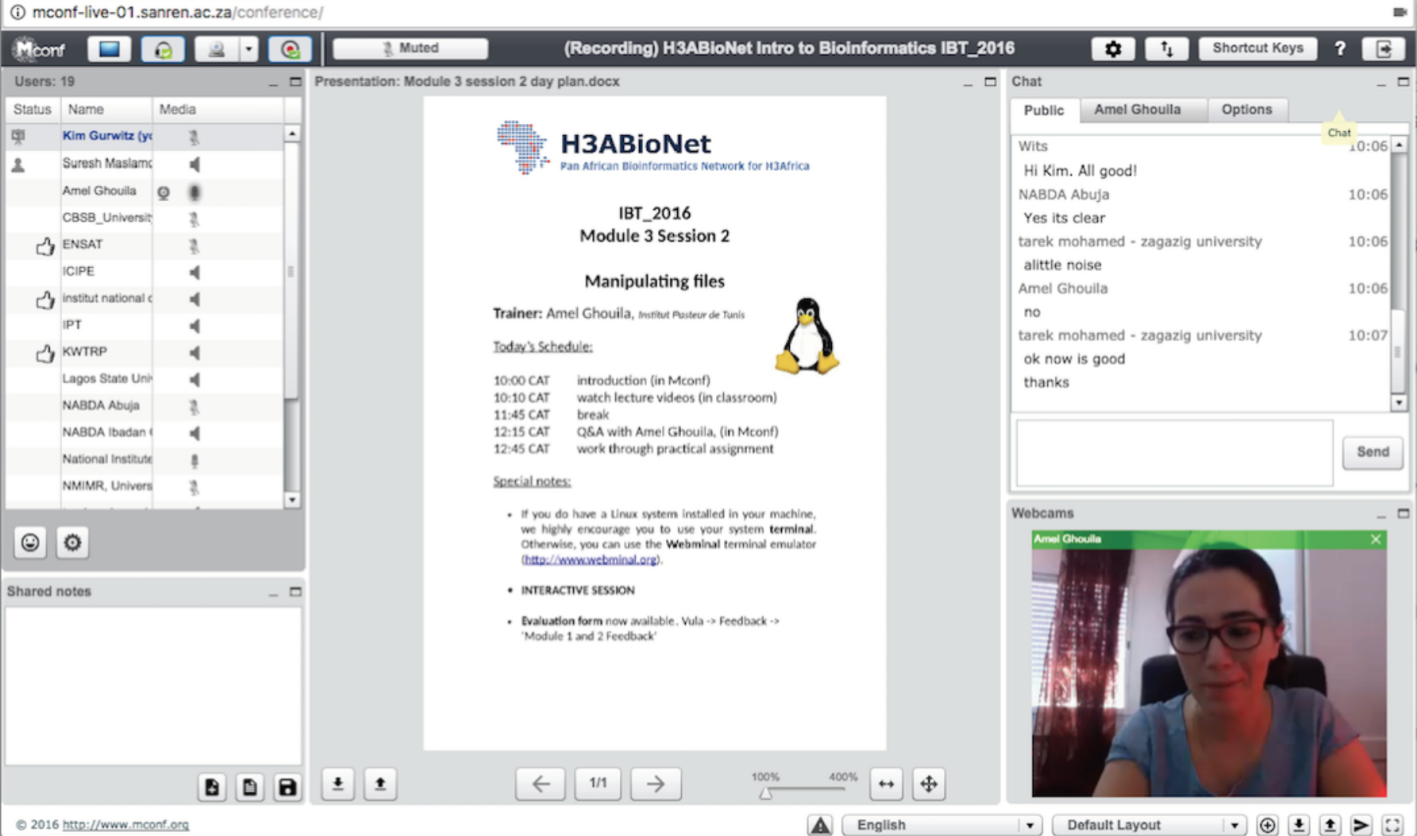
Virtual classroom using the Mconf web conferencing platform. Classrooms connected to the trainer and to other classrooms via the Mconf open-source web conferencing platform (http://mconf.org; in use here is the South African instance of Mconf https://mconf.sanren.ac.za/, hosted by SANREN http://www.sanren.ac.za/south-african-nren/). Classrooms either activated their microphones or entered text into a chat box to ask questions to the trainer. Trainers activated their webcams while answering questions. Trainers were able to upload their session resources in the central panel of the Mconf interface should they have wanted to explain a concept on a particular lecture slide, for example. Note: Consent to publish this image was obtained from the trainer shown in the figure. Abbreviation: SANREN, South African National Research Network.

During biweekly contact sessions (4 hours per day), classrooms connected with the expert trainer and with the other classrooms via the Mconf platform (https://mconf.sanren.ac.za/) for live video conferencing sessions (Figs 1 and 2). During these sessions, time was allocated for questions and answers with the trainer (Fig 2). These sessions also provided an opportunity for interaction across classrooms—classrooms enjoyed activating their webcams and waving to their colleagues across Africa. In this way, face-to-face elements were incorporated into distance learning components of the course in order to put a “face to the voice/screen” and to remind everyone that they belonged to an Africa-wide community comprising real people and real scientists across the continent (see section entitled “OER” for more on encouraging interactivity across classrooms).

#### Pre-recorded lectures

**Fig 3:**
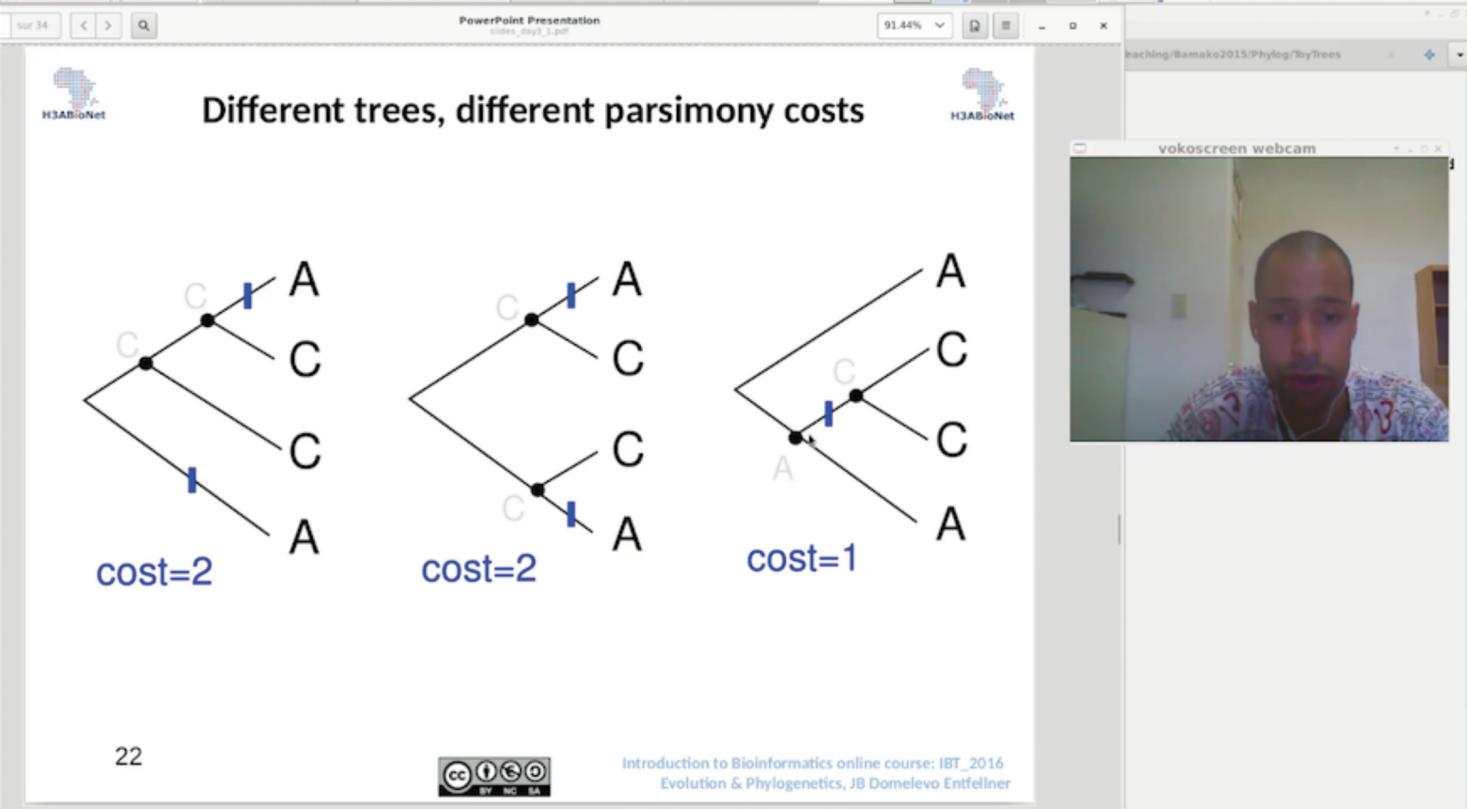
Pre-recorded lectures. Screenshot of a pre-recorded lecture with webcam activated. Note: Consent to publish this image was obtained from the individual in the figure.

In addition to live video conferencing during contact sessions, local classrooms watched pre-recorded and pre-downloaded lecture videos prepared by expert trainers via a locally set up projector system (Fig 3). The pre-recorded lecture videos were each restricted to roughly 15 minutes in length, which helped in reducing the size of the lecture videos to be downloaded with low internet bandwidth. In this way, unstable internet access was not a barrier to accessing lecture content. Short videos also allowed for a “mental breather” between concepts or groups of concepts for participants watching the videos. Tools used to create and register these recordings included freely available resources: Screencast-O-Matic (https://screencast-o-matic.com/) and the free, Libre, and open-source software VokoScreen (http://www.kohaupt-online.de/hp/). Trainers were encouraged to activate their webcams while recording their lectures because participants seemed to be motivated by being able to see their trainer while learning (Fig 3). This was also true for virtual classroom sessions during which webcams were activated (whenever bandwidth would allow) (Fig 2). Education scientists have established that nonverbal communication (e.g., watching someone’s facial expressions and body language) enhances the learning experience [10]. It is unclear from the IBT course data whether seeing the trainer assisted with learning, but it definitely seemed to increase attention and enjoyment of the contact sessions. An additional benefit of making the lecture recordings available before the contact sessions was that it allowed participants and staff to become familiar with the material before class. The availability of pre-recorded lectures to participants and staff prior to each contact session also provided the initial step for the possible adoption of a flipped classroom model for future iterations of the course [11]. The flipped classroom model would entail participants viewing the course material before the contact sessions and instead using the contact time to facilitate discussion and questions. If implemented in further iterations of the course, local staff would need to be adequately trained on the approach.

### OER

Incorporating OER into our learning model and, in particular, making these available online allowed for redundancy because it ensured that there was always more than one way to access course content and assistance. For example, even if a physical classroom could not connect to the virtual classroom due to internet instability, they could still access the course content via the course website (http://training.h3abionet.org/IBT_2016/). Furthermore, open-source web-based bioinformatics tools were used to ensure that access to particular software was not a barrier to education (see section entitled “Web-based tools”). The IBT training material is a valuable resource that can be utilized by the wider bioinformatics community, and the material will be reviewed and submitted to the Global Organisation for Bioinformatics Learning, Education & Training (GOBLET, http://www.mygoblet.org/).

#### Course management platform, Vula

**Fig 4:**
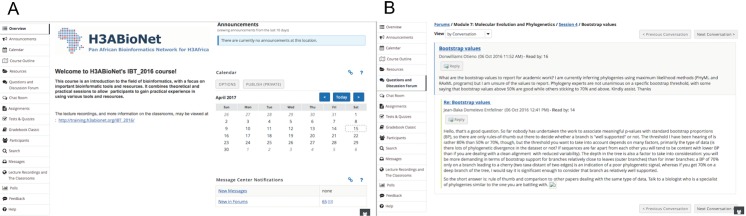
Screenshots of Vula site for IBT_2016 course. (A) Landing page of course site showing all tools. (B) Forums tool specifically showing the number of people that read the response to a particular question. In this example, at the time that the screenshot was obtained, 14 people had read the question, and 12 people had read the answer. Please note: Consent to publish this screenshot was obtained from participants of the relevant discussion. Abbreviation: IBT, Introduction to Bioinformatics.

The course management platform, Vula (http://vula.uct.ac.za), was utilized in order to send out announcements, manage participants, track their progress, and allow for live or delayed interaction amongst participants and with trainers and staff (Fig 4A). Vula is the University of Cape Town’s (UCT) online learning and collaboration environment built on Sakai, which is a free, open-source educational software platform (https://sakaiproject.org). A UCT member can create a project site, and guest accounts for that site can be created for any non-UCT members free of charge as long as the project is not for profit. Participants were able to log in with their unique credentials and could access, complete, and upload practical assignments and assessments for grading; each contact session had a practical component and practical assignment for hand-in, and each module had an end-of-module assessment comprising both theoretical and practical questions. In order to be eligible for the course letter of completion (i.e., course certificate), participants had to submit at least 90% of practical assignments and obtain a minimum overall mark of 60% in the assessments.

In addition, interactions between participants, trainers, and staff were facilitated by the question and discussion forums on the Vula site (Fig 4B). The forums allowed for the creation of a curated repository of questions and corresponding answers, which could be accessed by all members at any time even after the course was concluded. These forums were especially useful for asking questions outside of contact session time or for those that could not connect to the virtual classroom during contact sessions due to internet instability. The chat room on the Vula site allowed for informal interaction amongst participants from the various classrooms across Africa and created a friendly online atmosphere. Links to the lecture resources (lecture slides and pre-recorded lecture recordings, etc.) were also available via this site. Following each module, participants and staff members were able to access and anonymously complete an evaluation form on Vula in order for the course organizers to gain an impression of the quality and relevance of the content of each module.

#### Web-based tools

To further decrease barriers to education, trainers were encouraged to use web-based tools in their practical assignments to ensure that software limitations and computing infrastructure were not a disadvantage. H3ABioNet has also invested in installing eBioKits at some of the partner nodes to facilitate training activities (http://www.ebiokit.eu/). The eBioKit is a standalone device that can run bioinformatics analyses independent of the internet. Although the use of the eBioKit is an efficient approach to overcome the challenge posed by internet instability, at the time the course was designed, not all nodes had access to an eBioKit, and not all IBT classrooms are H3ABioNet nodes. This would be a limiting factor restricting some classrooms from benefitting from the IBT course, and therefore eBioKits are not currently used for the course. Using web-based tools also kept the classroom setup as simple as possible and enabled participants to access the tools via their own computers at home or in another computer laboratory. While internet instability was a challenge at certain classroom sites, deadlines were shifted accordingly to accommodate any campus-wide internet issues, thereby enabling participants to complete and submit assignments by the stipulated due dates.

## Lessons learned

Core features identified as crucial to the success of the IBT course in 2016, as well as areas to strengthen going forward, are outlined below. While we agreed to keep the results of the course evaluation confidential, the following points were evident to the course organizers and in conversations with participants and volunteer staff.

### What worked?

#### Expert trainers

One of the biggest pillars of success of the IBT course was the quality and experience of the expert trainers. Apart from a guest introductory lecture given by expert European bioinformaticians, all our trainers are based within Africa, and most are H3ABioNet members. They provided excellent lectures and content, set weekly practical assignments, and embraced a new course design model that fell, for most, outside of their comfort zone.

#### Volunteer staff in local classrooms

A course of this design and size would not have been possible without dedicated local volunteer staff—the teaching assistants, system administrators, and principle investigators. Local staff were key in securing and setting up their training rooms and in ensuring that participants kept up to date with the various course requirements in order to pass the course. As several of the volunteer staff were PhD candidates or postdoctoral students—many of whom will go on to teach in some form or another during their careers—this course provided a platform for education-capacity development. We hope that this will benefit participating institutions in future introductory bioinformatics training endeavors and perhaps in more advanced bioinformatics training courses, too. Short biographies of the local staff for each of the IBT_2016 classrooms can be viewed on the IBT_2016 course website (http://training.h3abionet.org/IBT_2016/?page_id=130).

#### Redundancy

Another key feature of the course is redundancy in course delivery methods—there was always more than 1 avenue through which to access the course content and to access assistance. Internet stability can be a challenge anywhere in the world and is exacerbated in many African regions. By ensuring redundancy (e.g., live question and answer sessions via video conferencing sessions as well as question and discussion forums on the course management platform), we could ensure that internet instability was not a barrier to education. Crucially, providing pre-recorded lectures ensured that classrooms could continue with the course locally during contact sessions even if they could not connect to the virtual classroom on a particular day.

#### Communication and interaction across and within classrooms

This course provided a unique opportunity for connecting young scientists across Africa. Interacting in the virtual classroom and in the online forums and chat rooms greatly enhanced the learning experience for all. Linking the local classrooms during the regular contact sessions not only provided the opportunity for junior scientists to develop new skills but also created an environment to facilitate future collaborations between African scientists. Furthermore, interaction among peers and with teaching assistants within physical classrooms provided great support and motivation for the participants.

### What could be strengthened?

In addition to continuing to advocate for and strengthen the above points, there are certain additional elements that would benefit a course like IBT.

#### Staff training

While staff were supported by the core team throughout the course, providing formal training would prepare the local staff for the running of their classroom by providing a space for the staff to establish a team atmosphere in which everyone knows their responsibilities and how they might assist each other during the course, providing facilitation skills training so that they may adequately assist participants, and providing training on the various platforms used in the course. In this way, training capacity is increased at the local institutions to serve future training endeavors. Staff training has been implemented in the 2017 iteration of the course.

#### Community engagement

Drawing participants’ attention to bioinformatics research and activities taking place in their own institution might inspire collaborations and ensure that participants utilize the skills learnt in the course, perhaps enabling them to progress in their bioinformatics abilities. The IBT course provides a wonderful platform to facilitate this engagement and to bring together molecular biologists and bioinformaticians.

## Looking forward

IBT_2016 proved a successful endeavor, with 195 participants (53%) meeting all the course requirements and receiving a letter of completion for the course. The course is running again in 2017 (https://training.h3abionet.org/IBT_2017/). We have incorporated the lessons learnt in the new iteration of the course and opened the course to a wider African research community. We are also looking at the possibility of running additional short courses using this model and are running an “Introduction to Genomic Medicine for Nurses” course for 17 weeks (https://training.h3abionet.org/AGMC_2016/). We would like to reiterate that, with a bit of creativity and a lot of dedication, this type of initiative can work even in a resource-limited setting; the IBT course is a testament to this.

## Supporting information

S1 File Detailed description of the different modules provided in the IBT_2016 course, including learning objectives and learning outcomes. (PDF)
