# Supplementary material for: Designing a course model for distance-based online bioinformatics training in Africa: The H3ABioNet experience
Source: PLoS Comput Biol. 2017 Oct 5;13(10):e1005715. doi: 10.1371/journal.pcbi.1005715 (PMC5628786; doi:10.1371/journal.pcbi.1005715)
Supplement: S1 File — (PDF) [file pcbi.1005715.s001.pdf]

H3ABioNet

Pan African Bioinformatics Network for H3Africa

H3ABioNet Introduction to Bioinformatics 2016 online course: IBT_2016
Learning Objectives and Learning Outcomes

This document details the learning objectives and learning outcomes that participants of the H3ABioNet 'Introduction to Bioinformatics 2016' course (IBT_2016) should acquire by the end of each module. It is intended to inform the development of detailed module content and assessments.

This document was developed by the trainers of the IBT_2016 course (http://training.h3abionet.org/IBT_2016/?page_id=1007)

Definitions:

Learning objective: knowledge learned without implementation
(content covered in the lecture component of contact sessions)

vs.

Learning outcome: measurable - skills gained
(reinforced and practised in the practical assignment component of contact sessions)

Please note, up to 4 hours are allocated per contact session: 3 hours teaching time, 30 minute break, 30 minutes misc in case of technical difficulties. Each session comprises a lecture and practical component. Trainers may decide how best to divide the teaching time.

Module Name: Introduction to bioinformatics and bioinformatics resources and databases

Final resources for the 2016 version of this module can be found here:
http://training.h3abionet.org/IBT_2016/?page_id=30

Prerequisites

- Basic background in molecular biology
- Basic use of an internet browser

Curriculum

Session 1: What is Bioinformatics and why is it important?

Learning objectives and outcomes

- Provide an introduction to what bioinformatics is and why it is important
- Provide an overview of the application areas of bioinformatics, with a focus on the topics that will be taught in the course
- Explain what type of knowledge will be gained from the course

Session 2: Biological databases and resources (NCBI, EBI)

Learning objectives:

- Describe how bioinformatics data is stored and organised
- Describe the different types of data found at the NCBI and EBI resources
- Explain how to locate and extract data from key bioinformatics databases and resources

Learning outcomes:

- Locate and use the main databases at the NCBI and EBI resources
- Know the difference between databases, tools, repositories and be able to use each one to extract specific information
- Extract data from specific databases using accessions numbers, gene names etc.

- Use selected tools at NCBI and EBI to run simple analyses on genomic sequences

<u>Session 3:</u> Advanced literature searching – PubMed

Learning objectives:

- Outline the background and framework of PubMed
- Define the logic of breaking down search queries into keywords and constructing advanced search queries
- Describe the basic search tags and boolean operators used for generating specific search queries on PubMed
- Outline the approach used to build an advanced search query in PubMed and further refine search results

Learning outcomes:

- Identify keywords to be used to generate a seach query
- Locate and use the advanced search query page on PubMed
- Use the advanced search page on Pubmed to generate complex search queries using provided examples
- Use some of the major search fields (tags) to generate specific search results and further refine search results for a specific topic of interest

<u>Session 4:</u> DNA analysis – e.g. sequence features, start site, restriction sites etc

Learning objectives:

- Describe a selection of the simple features that can be annotated on a DNA sequence of interest
- Describe how to extract a DNA sequence from a database
- Outline a selection of simple tools used to annotate features on a DNA sequence

Learning outcomes:

- Extract a DNA sequence and save it in the correct format

- Identify features on the sequence such as coding regions, restriction enzyme sites, etc.
- Interpret sequence analysis results and what functional regions mean biologically

Module Name: Genomics

Final resources for this module can be found here:
http://training.h3abionet.org/IBT_2016/?page_id=644

Prerequisites
- Basic background in molecular biology
- Basic genetics
- Basics of using internet

Curriculum

Session 1: Genome sequences – sequencing technology and NGS overview

Learning objectives:
- Outline the brief historical context of sequencing and genomics - from Sanger to NGS, human genome project, hapmap, 1000 genomes, UK10K etc in brief
- Explain the different NGS study designs
- Differentiate between the various NGS sequencing technologies
- Outline the DNA-seq analysis pipeline and associated file formats

Learning outcomes:
- Describe the details of the Illumina sequencing technologies
- Explain the steps of the DNA-seq analysis pipeline
- Describe information contained in the various file formats produced by the DNA-seq analysis pipeline - SAM and vcf files

- Navigate through database repositories to retrieve, extract and use NGS datasets

<u>Session 2:</u> **Genome browsers and annotation**

Learning objectives:

- Outline the need for genome browsers and define what they are
- Describe the genome annotation pipeline, including manual and automated annotation
- Demonstrate how to find genomic information and features using genome browsers
- Demonstrate how to retrieve data from genome browsers using Ensembl's BioMart as an example

Learning outcomes:

- Use the Ensembl genome browser to explore the available annotation tracks for a gene of interest
- Use BioMart to retrieve a customised data set based on a biological question of interest

<u>Session 3:</u> **Comparative Genomics**

Learning objectives:

- Outline the application areas of comparative genomics
- Discuss how the availability of whole genome sequences and computational tools has led to comparative genomics being an integral field of genomics
- Examine some of the available tools that are based on comparative genomics - precomputed alignments in browsers such as Ensembl, Vista Browser, GERP conservation scores; functional predictions based on comparative genomics e.g. Sift and Polyphen

Learning outcomes:

- Use Ensembl to to explore the annotations for a gene of interest
- Explore the conservation of a gene across different organisms
- Contrast between paralogs and orthologs
- Describe the concept of synteny of a genomic region between species

Session 4: Human variation

Learning objectives:

- Outline the different types of human genetic variation
- Define linkage disequilibrium and haplotypes
- Provide a description of the variant call format (.vcf) file
- Provide examples of the available human variation databases and functional prediction tools

Learning outcomes:

- Extract specific information from a vcf file
- Extract gene specific SNP data from publicly available databases
- Annotate a .vcf file using ANNOVAR
- Explore allele frequency differences between populations
- Extract information for a variant of interest from disease related SNP databases

Module Name: Introduction to Linux

Final resources for this module can be found here:
http://training.h3abionet.org/IBT_2016/?page_id=915

Prerequisites

- None

Curriculum

Session 1: Introduction to Linux and UNIX and the command line

Learning objectives:

- Be aware of the Unix file structure and how to navigate through it
- Learn how to create and access files and directories
- Explain full and relative file paths and when to use them
- Knowledge of useful shortcuts, wildcards, tab completion

Learning outcomes:

- Be able to navigation through the Linux filesystem
- Be able to create new files and directories
- Be able to copy, move, rename and delete files
- Be able to view and navigate through open files using less, head, cat, tail

Session 2: Manipulating files. Useful commands and tips

Learning objectives:

- Explain the concept of files and how they are created in Linux
- Describe how to extract data from files using grep, cut, sort, uniq and output redirection

Learning outcomes:

- Be able to create and edit files using a text editor nano
- Be able to search through a file for a matching string or regular expression using grep
- Be able to use common commands such as cut, sort, uniq
- Be able to redirect the output of a command to a file

Session 3: Permissions, groups, and process control

Learning objectives:

- Explain file and directory permissions and how to change them
- Explain loops, variables and script generation to automate tasks
- Explain environment variables and why they are important
- Learn how to ssh onto a remote machine

Learning outcomes:

- Be able to view and change file and directory permissions
- Be able to generate variables to store information
- Be able to view and understand environment variables
- Generate loops to run a sequence of commands on multiple files
- Ssh onto a remote machine

Module Name: Sequence alignment theory and applications

Final resources for this module can be found here:
http://training.h3abionet.org/IBT_2016/?page_id=1028

Prerequisites

- Basic molecular biology
- Introduction to bioinformatics resources and databases (Module 1)

Curriculum

Session 1: Introduction to searching and sequence alignment

Learning objectives:

- List the concepts and applications of sequence searching
- Define the concepts of homology, identity, orthologues, paralogues
- Provide examples of basic sequence alignment, introducing concepts of point mutations, deletions, insertions etc.

- Provide an outline of the different approaches to sequence alignment - exhaustive vs. heuristic

Learning outcomes:

- Extract and generate pairwise sequence alignments for a protein sequence of interest
- Describe and interpret the metrics used to assess the quality of a pairwise sequence alignment, identity versus similarity
- Describe the differences between homologues, paralogues and orthologues
- Use a pairwise sequence approach to identify mutations between two sequences

<u>Session 2:</u> Pairwise sequence alignment

Learning objectives:

- Define the differences between global and local pairwise alignment algorithms
- Outline the basic principles of pairwise alignments, scoring matrices and gap penalties
- Describe the concept of the dynamic programming approach for pairwise sequence alignment (global and local)

Learning outcomes:

- Align two sets of sequences using both a global and local alignment approach
- Explain the effect of changing parameters such as scoring matrices, gap penalties etc.
- Interpret the output of a pairwise alignment and when the global or local alignment method is more appropriate to use

<u>Session 3:</u> BLAST algorithm

Learning objectives:

- Outline the principles of the BLAST algorithm
- Differentiate between the different BLAST algorithms, parameters, and their applications
- Define how the sensitivity and specificity of BLAST searches can be changed by adjusting parameters
- Generate and evaluate BLAST results

Learning outcomes:

- Select the correct BLAST algorithm and database for the appropriate biological question being asked, i.e. select between blastp, blastn, blastx etc.
- Interpret the the various BLAST output metrics/results
- Analyse results based on the various output metrics

Module Name: Multiple sequence alignment

Final resources for this module can be found here:
http://training.h3abionet.org/IBT_2016/?page_id=1165

Prerequisites

- Basics of molecular biology
- Sequence alignment theory and applications (Module 4)
- Basics of molecular genetics

Curriculum

Session 1: Application of multiple sequence alignment

Learning objectives:

- Outline the application areas for multiple sequence alignments
- List the main criteria for building a multiple sequence alignment
- Describe how to interpret multiple sequence alignments

Learning outcomes:

- Retrieve a protein sequence of interest for different organisms from Uniprot
- Generate a basic multiple sequence alignment using Clustal
- Assess the relationships between the protein sequences from the different organisms based on the multiple sequence alignment
- Identify possible conserved protein domains and amino acids.

Session 2: Choosing the right sequences with the right software

Learning objectives:

- Outline the methods used to visualise and edit multiple sequence alignments
- Introduce the Jalview software and describe the various features and functionality of the software
- Explain how to use Jalview to view, edit and save a multiple sequence alignment

Learning outcomes:

- Load a multiple sequence alignment generated using other tools into Jalview
- Navigate through the multiple sequence alignment using Jalview
- Edit the multiple sequence alignment using Jalview
- Save and export the edited multiple sequence alignment

Topic Name: Protein structural bioinformatics

Final resources for this module can be found here:
http://training.h3abionet.org/IBT_2016/?page_id=1235

Prerequisites
- Basic knowledge in biochemistry and/or molecular biology
- Databases and resources (Module 1)
- Sequence alignment theory and application (Module 4)
- Basics of molecular genetics

Curriculum

Session 1: Introduction to Protein Structure Bioinformatics

Learning objectives:
- Recap some basics about amino acids and proteins.
- Outline the different levels and organisation of protein structures.
- Describe how protein structures are determined.
- Describe some of the relevant protein databases including PDB and associated file formats and file viewers.

Learning outcomes:
- Use the appropriate tools to predict the secondary structure for a protein sequence of interest
- Query PDB with a sequence of interest to identify homologous protein sequences
- Use PDB to extract information relevant to a protein structure of interest.

Session 2: Comparative Protein Structure Modeling

Learning objectives:
- Describe the principles of protein structural prediction
- List the resources available for protein structure prediction
- Explain the application of predicting protein structures
- Define the criteria for assessing and refining a predicted protein structure
- Know how to search for homologous protein structures (PSIPRED)

- Predict 3D structures for proteins using MODELLER and SwissModel
- Assess the quality of 3D structures

Learning outcomes:

- Search for and identify homologous protein structures/template
- Predict the structure for a protein sequence based on an identified template
- Determine if the correct fold has been assigned to a model
- Distinguish between good, medium, and poor quality structures

Session 3: Molecular Docking

Learning objectives:

- Describe the process and principles of molecular docking
- Be aware of the PatchDock algorithm
- Interpret PatchDock results

Learning outcomes:

- Perform an *in silico* docking experiment
- Interpret the geometric and ACE score
- Compare predicted vs experimental pose
- Assess if the docking parameters are optimised

Topic Name: Molecular evolution and phylogenetics

Final resources for this module can be found here:
http://training.h3abionet.org/IBT_2016/?page_id=1256

Prerequisites

- Basic familiarity with biological sequences (DNA and protein)

Curriculum

Session 1: Historical perspective on the development of the theory of evolution. Mechanisms of molecular evolution and speciation

Learning objectives:

- Know about the genesis of the theory of evolution.
- Know about the mechanisms involved in molecular evolution and speciation
- Have an understanding of how the scientific concepts of species and evolution gradually matured over the 18th, 19th and 20th centuries
- List the key contributions by Charles Darwin
- List the main driving forces of evolution at organism and molecular levels
- State the standard definition of a species and the processes leading to the emergence and extinction of species
- Think in terms of populations rather than individuals, and understand that is the appropriate scale for evolutionary studies

Learning outcomes:

- Explain the processes of biological evolution and speciation
- Use skills of taxonomic observation to "see" adaptive evolution at work
- Describe the famous species of finches Darwin observed during his journey to the Galápagos islands on the *Beagle*

Session 2: Building phylogenies from biological data nowadays: evolutionary distances between sequences, models of DNA and amino acid evolution

Learning objectives:

- Outline the types of data used to build phylogenetic trees. Understand the concepts of phylogenetic distance and how models of evolution are used
- Explain the difference between discrete, semi-continuous and continuous traits.

- Contrast between observed distance and evolutionary divergence time between sequences
- Be aware of the Markovian property and time-reversibility property
- Explain the idea of independence of sites and the concept of phylogenetic signal in an alignment. Be aware of the need to pre-process the alignment by trimming low-signal regions of the alignment.
- Know about some common DNA and amino-acid models of evolution (JC69, GTR, WAG)

Learning outcomes:

- Manipulate a nucleotide alignment and run phylogeny jobs on a web server.
- Manipulate a simple nucleotide alignment in Fasta format and extract basic information from it
- Interact with the phylogeny.fr servers, submit jobs and retrieve results
- Discover the Newick/New Hampshire format for trees
- Run basic jobs with PhyML on phylogeny.fr
- Compare HKY85 and GTR models of nucleotide substitution

<u>Session 3:</u> **Complexity of the space of phylogenetic models, heuristics to map it. Tree building with distance matrix methods. Parsimony approaches**

Learning objectives:

- Define the properties of phylogenetic trees, methods to optimize the topology and branch lengths of a tree. Distance matrix and parsimony approaches
- Explain that the search space of phylogenies is huge.
- Describe basic combinatorial properties of trees and the necessity to develop heuristics to search efficiently for the tree maximizing a criterion
- Explain how to build phylogenies from pairwise distance matrices (UPGMA and NJ algorithms)
- Explain the concept of Minimum Evolution (Maximum Parsimony) and how to use it to infer trees

Learning outcomes:

- Use distance matrix methods for phylogenetic reconstruction and Maximum Parsimony approaches
- Be able to build “by hand” an UPGMA tree from a (5x5) distance matrix
- Run BioNJ reconstructions on phylogeny.fr
- Run Maximum Parsimony inferences with the TNT software on phylogeny.fr

<u>Session 4:</u> **Tree building with maximum likelihood approaches. Bayesian viewpoint. Branch support measures**

Learning objectives:

- Define what likelihood is, and the principle of maximum likelihood inference.
- Explain what the prior and posterior of a model is with respect to bayesian approaches.
- Describe why confidence values need to be calculate for the branches of a tree.
- Explain Bayesian inference with respect to tree building

Learning outcomes:

- Manipulate real-world DNA alignments (mitochondrial DNA), and run Maximum Likelihood inferences and Bayesian inferences
- Be able to run PhyML jobs with different models of evolution and interpret their results, using the online platform phylogeny.fr
- Compare results with those obtained through Maximum Parsimony approaches
- Analyse the results of bootstrap support computations on branches of an ML phylogeny
- Run Bayesian analyses with MrBayes (still via job submission on phylogeny.fr)
